# Effect of Increased Influent COD on Relieving the Toxicity of CeO_2_ NPs on Aerobic Granular Sludge

**DOI:** 10.3390/ijerph16193609

**Published:** 2019-09-26

**Authors:** Xiaoying Zheng, Yuan Zhang, Wei Chen, Weihong Wang, Hang Xu, Xiaoyao Shao, Mengmeng Yang, Zhi Xu, Linghua Zhu

**Affiliations:** 1 Key Laboratory of Integrated Regulation and Resource Development on Shallow Lake of Ministry of Education, College of Environment, Hohai University, Nanjing 210098, China; zhangyuan8355@163.com (Y.Z.); cw5826@hhu.edu.cn (W.C.); xuhang810826@163.com (H.X.); xyshao727@163.com (X.S.); Yangmm1021@163.com (M.Y.); xuz_hhu@163.com (Z.X.); zhulhwx@163.com (L.Z.); 2 College of Hydraulic and Civil Engineering, Xinjiang Agricultural University, Nongda East Road No. 311, Sayibak District, Urumqi 830052, China; wangwh95515@163.com

**Keywords:** CeO_2_ nanoparticles, stratified extracellular polymeric substances, aerobic granular sludge, recovery

## Abstract

Due to the increased use of cerium oxide nanoparticles (CeO_2_ NPs), their potential environmental risks have caused concern. However, their effects on the aerobic granular sludge (AGS) process and the later recovery of AGS are still unclear. In this study, we comprehensively determined the changes in pollutant removal and the levels of extracellular polymeric substances (EPS) in AGS that were exposed to CeO_2_ NP treatments (0 (the control, R0), 1 (R1), and 5 (R5) mg/L), following an increase in the influent chemical oxygen demand (COD). An increase in the CeO_2_ NP concentration enhanced their inhibitory effect on the removal of total nitrogen (TN) and total phosphorus (TP), and promoted the production of polysaccharides (PS) and proteins (PN) in loosely bound EPS (LB-EPS) or tightly bound EPS (TB-EPS), as well as the dissolved organic carbon (DOC) components in EPS, but had no long-term effects on the removal of organic matter. When the addition of CeO_2_ NPs was stopped and the concentration of influent COD increased, the TN and TP removal efficiencies in R1 and R5 slowly increased and recovered. In R1, they were only 4.55 ± 0.55% and 2.71 ± 0.58% lower than in R0, respectively, while the corresponding values for R5 were 5.06 ± 0.46% and 6.20 ± 0.63%. Despite the LB-EPS and TB-EPS concentrations in the R1 and R5 treatments recovering and being similar to the levels in the control when no CeO_2_ NPs were added, they were still slightly higher than in the R0, which indicating that the negative effects of CeO_2_ NPs could not be completely eliminated due to the residual CeO_2_ NP levels in AGS.

## 1. Introduction

Cerium oxide nanoparticles (CeO_2_ NPs) have been widely used in a diverse range of commercial and scientific fields, such as textiles, pharmaceuticals, catalysis and catalyst supports, personal care products, and biomedical products, due to their novel physicochemical properties [1]. Inevitably, the increasing use of products containing CeO_2_ NPs has resulted in their discharge into sewer systems during their manufacturing and disposal, from where they finally enter municipal wastewater treatment plants (WWTPs), which face the greatest exposure and act as the final barrier that prevents NPs from entering the aquatic environment [2,3]. As a consequence, these particularly tiny particles, with unique properties, could lead to biological toxicity in microorganisms and reduce the effectiveness of wastewater treatment, which eventually causes considerable risks to the ecological environment [4,5]. The production of CeO_2_ NPs is almost one thousand tons per year [6], and this figure is expected to rapidly increase as the industry develops. Different quantities of CeO_2_ NPs are present in air, surface water, and sewage [7], in which the concentration can reach about 0.1 mg/L [8]. Cerium oxide NPs can have adverse effects on bacteria [9], change the structure of biofilms [10], decrease the microbial diversity of activated sludge [11], and generate acute toxic effects on aquatic life or human lung cells [12]. When NPs enter WWTPs, large amounts can be adsorbed and removed from wastewater during the treatment process [13]. During the biological treatment processes in WWTPS, adsorbed NPs can produce varying degrees of cytotoxicity in microorganisms, which results in short or long-term environmental risks [4,5,14]. There is an urgent need to assess the environmental impacts of CeO_2_ NPs on WWTPs.

Biological cells and some other organic or inorganic substances can be intertwined and aggregated into a compact form, which do not need a firm carrier in a sequencing batch reactor (SBR). The intertwined granules have high biomass, excellent settling behavior, and considerable resistance to the various degrees of toxicity in wastewater [15]. Aerobic granular sludge (AGS) is one such microbial aggregate, which has the properties of high energy effectiveness, limited nutrient requirements, and low sludge production [16]. The aggregated sludge system is a species-rich ecosystem, where a variety of bacteria live together and achieve different metabolic functions, such as the removal of organics and nutrients, through various interactions [17,18]. Extracellular polymeric substances (EPS), which are produced by microbes, act as a special gel-like matrix that twines the microbial cells together to construct more complex polymers, and can protect microorganisms from the changeable and harsh external environment [19,20]. The EPS can be divided into outer and inner regions according to their location, and are referred to as loosely bound EPS (LB-EPS) and tightly bound EPS (TB-EPS), which form two stratified protective barriers [3]. Polysaccharides (PS) and proteins (PN), which are the main EPS, gather around the outer layer of cells, helping granular sludge to survive from exposure to iron oxide NPs [21]. When exposed to CeO_2_ NPs, the PN concentration in EPS has been shown to increase due to production by activated sludge [22], and the production and breadth of PS in wastewater biofilms also increases to form a formidable shield [23]. Therefore, EPS are essential in the stabilization of AGS when exposed to NPs. However, there is still a limited understanding of the distribution of PS and PN in EPS when the AGS recovers following the removal of CeO_2_ NPs.

In this study, AGS was cultivated in SBRs to determine the influence of CeO_2_ NPs on AGS and toxicity alleviation in the absence of CeO_2_ NPs. In the first stage (30 d), we decreased the nutrient levels in synthetic wastewater to simulate real municipal sewage. In the second stage (30 d), we increased the influent chemical oxygen demand (COD) to help AGS to relieve the toxicity of CeO_2_ NPs, which were added in the first stage. The main objectives were as follows: (i) to investigate how the increased influent COD helped AGS recover its nitrogen and phosphorus removal efficiencies when no CeO_2_ NPs were added; (ii) to explore the changes in the LB-EPS and TB-EPS concentrations, as well as the PS and PN within them at different CeO_2_ NP concentrations, and therefore better understand how EPS protect AGS from CeO_2_ NP toxicity and help AGS to recover in the absence of CeO_2_ NPs; and, (iii) to determine the variations of dissolved organic carbon (DOC) components in different stages by liquid chromatography with an organic carbon detection (LC-OCD) system.

## 2. Methods

### 2.1. Set-up and Operation

**Figure 1:**
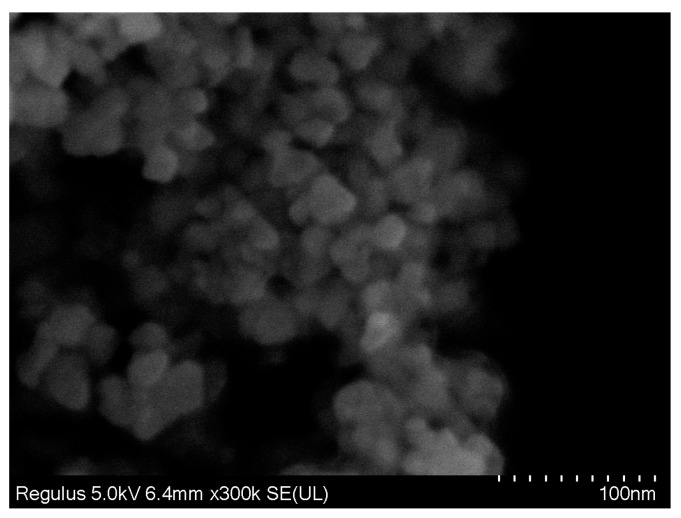
Scanning electron micrograph (SEM) image of cerium oxide nanoparticles (CeO_2_ NPs) used in this study.

Cerium oxide NPs were purchased from the Shanghai Aladdin Bio-Chem Technology Company, LTD (Shanghai, China). The average size was 40 nm and the density was 6.71 g/mL at 25 °C. The surface morphology of the CeO_2_ NPs was examined with a scanning electron microscope (SEM, Hitachi Regulus SEM) (Figure 1). A 100 mg/L stock solution of CeO_2_ NPs was prepared through a method, in which 100 mg of CeO_2_ NPs were added to 1 L of Milli-Q water, followed by 1 h of ultrasonication (25 °C, 120 W, 40 kHz) [24].

In this study, 1 and 5 mg/L CeO_2_ NPs were chosen as the two test concentrations. 1 mg/L CeO_2_ NPs was chosen as the lower level, which was typical of the environmental concentrations of CeO_2_ NPs entering WWTPs, because WWTPS are responsible for preventing nanomaterials from entering the environment [10]. A higher level of 5 mg/L CeO_2_ NPs was also investigated in recognition of the likely more extensive future production and application of CeO_2_ NPs [6].

Three SBRs, with CeO_2_ NP concentrations of 0 mg/L (control reactor, R0), 1 mg/L (R1), and 5 mg/L (R5) in synthetic wastewater, were operated in parallel for 60 d (two stages). In the first stage (30 d), 0, 1, and 5 mg/L CeO_2_ NPs were added into the influent of the three reactors. In the second stage (30 d), we stopped adding CeO_2_ NPs into the reactors, and increased the influent COD. The operational modes of the two stages are shown in Table 1. The working volume of each SBR was 8 L, with an internal diameter of 160 mm and height of 400 mm (H/D = 2.5). The volumetric exchange ratio was 60%. The total cycle time was 6 h: 20 min. for anaerobic feeding, 90 min. for the anaerobic period, 130 min. for the aerobic period, 110 min. for the anoxic period, 1 min. for sludge settling, and 9 min. for effluent discharge and idling. The reactors continuously ran and did not stop between two batch reactions, so the reactors performed 240 times over the 60 d total run time. The temperature was controlled to 21 ± 4 °C and the pH was maintained between 6.5 and 8.5.

The AGS that was used in the experiments was obtained through two-months of laboratory culture of activated sludge that was taken from a secondary sedimentation tank in a municipal wastewater treatment plant, and their average particle diameter was 1.40 mm. The initial mixed liquor suspended solids (MLSS) concentration in each reactor was 5 g/L. The AGS that was used in this experiment was cultured with synthetic wastewater, whose components were as follows (mg/L): COD, (C_6_H_12_O_6_ and CH_3_COONa), 175 for 30 days and 350 for 30 days; ammonia nitrogen (NH_4_^+^–N), (NH_4_Cl), 40; PO_4_^3^^−^–P, (KH_2_ PO_4_), 5; CaCl_2_, 70; MgSO_4_, 28; and, FeSO_4_, 28. Trace elements in the AGS were also indispensable for microbial growth [25].

### 2.2. Extraction and Analysis of EPS

The samples of AGS were removed from the three SBRs every ten days to determine the stratified EPS. The LB-EPS and TB-EPS were extracted with the centrifugation and cationic resin extraction method [3].

The LB-EPS and TB-EPS concentrations were analyzed to determine the PS and PN concentrations. Zheng et al. described the method used to analyze the soluble PS and PN concentrations in EPS [25].

### 2.3. Evaluation of EPS Components by LC-OCD

The LC-OCD system (Model 9, DOC-LABOR Dr. Huber, Germany) was used for determining the fraction of organic matter in extracted stratified EPS [26]. The concentrations of biopolymers, building blocks, low-molecular weight (LMW)-acids, and LMW-neutrals in terms of the DOC were determined through the LC-OCD system. Briefly, an on-line purified mobile phase (a phosphate buffer (22.5 mmol), pH 6.59 exposed to UV-Oxidation in the DOCOX-reactor) was delivered to an autosampler with a high performance liquid chromatography (HPLC) pump (Azura P 4.1S, Knauer, Germany) at a rate of 50 mL/min. (MLE, Dresden, Germany, 1000 μL injection volume) and a chromatographic column (250 × 20 mm, TSK HW 50S, 3000 theoretical plates, Toso, Japan). Approximately 10% of the sample bypassed the column and was directly analyzed as DOC. After passing through the column the liquid entered a UV-detector (UVD) where measurements were made at 254 nm (Azura UVD 2.1S, Knauer). After the UVD, the flow separated and the main volume entered the organic-carbon-detector (OCD), together with phosphatidic acid solution (as acidification solution) and nitrogen (as carrier gas).

### 2.4. Other Analytical Methods

The COD, NH_4_^+^–N, TN, TP, and mixed liquor volatile suspended solids (MLVSS) concentrations were measured with standard methods [27].

Most of the experiments were conducted in triplicate, and the results were presented as the mean ± standard deviation. An analysis of variance (ANOVA) was conducted, with statistical significance accepted at *p* < 0.05. 

## 3. Results and Discussion

### 3.1. Sludge Properties and the Production of EPS in AGS

As a microbial aggregate, AGS has a high biomass content and the EPS produced by microbes, acts as a gel-like matrix, which twines the microbial cells together to construct more complex polymers. Therefore, biosorption is the major physical removal mechanism by which AGS removes CeO_2_ NPs, which results in many NPs accumulating on the surface of AGS.

**Figure 2:**
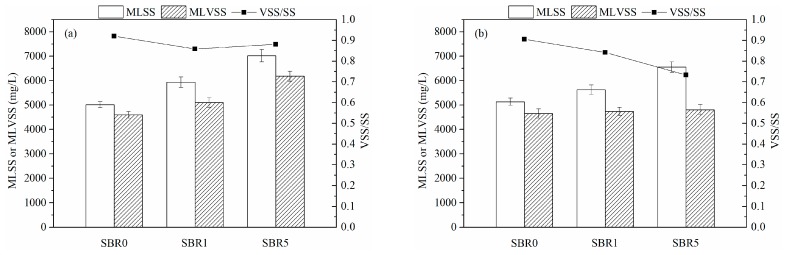
Mixed liquor suspended solids (MLSS) and mixed liquor volatile suspended solids (MLVSS) concentrations in aerobic granular sludge (AGS); (**a**) the first stage (the 30th day), and (**b**) the second stage (the 60th day).

The MLSS and MLVSS concentrations are important indicators of sludge performance. The MLSS and MLVSS were examined on the 30th and 60th days of the experiment to explore the sludge properties of AGS and its recovery after continuous exposure to CeO_2_ NPs, with the results shown in Figure 2. In the first stage, due to the adsorption of inorganic materials (CeO_2_ NPs), with a high specific gravity in the sludge, the MLSS concentration in the AGS fed with CeO_2_ NPs increased. The MLSS concentrations in the R1 and R5 treatments were higher at 5940 ± 126 and 7020 ± 254 mg/L, respectively, when compared with the control (5010 ± 126 mg/L). Figure 2 shows that the biomass content in the R1 treatment was slightly higher than in the control, while the biomass in the R5 treatment was 6084 ± 203 mg MLVSS/L, which was significantly higher than the control (4600 ± 136 mg MLVSS/L). These data indicated that when AGS were exposed to CeO_2_ NPs, more biomass was produced to maintain the stability of reactor performance. In the second stage, no more CeO_2_ NPs were added into the reactors. The MLSS and MLVSS concentrations in the control were relatively unaffected despite the influent COD being increased from 175 to 350 mg/L, being maintained at 5136 ± 145 and 4650 ± 187 mg/L, respectively. When compared to the first stage, the MLSS concentrations in the R1 and R5 treatments decreased to 5630 ± 197 and 6560 ± 218 mg/L, respectively, which were still higher than in the control, because the adsorbed NPs could not escape from the reactors. However, on the 60th day, the MLVSS concentrations in the R1 and R5 treatments basically decreased to the same concentration as in the control. The CeO_2_ NPs that had been added in the first stage had been encapsulated by some organic or inorganic substances, and therefore had few negative effects in the second stage.

**Figure 3:**
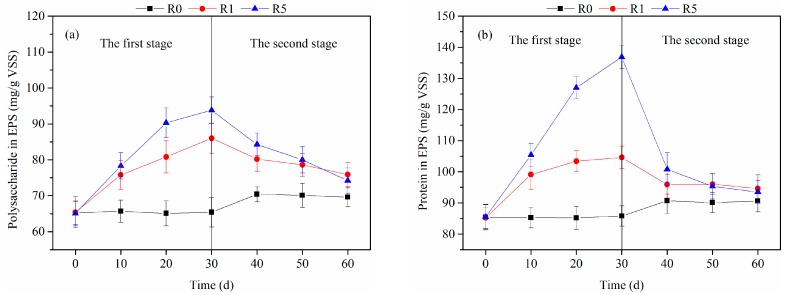
Effects of cerium oxide nanoparticles (CeO_2_ NPs) on (**a**) polysaccharide (PS) and (**b**) protein (PN) production in extracellular polymeric substances (EPS).

Figure 3 shows the production of PS and PN in the EPS secreted by AGS during the 60 days. In the first stage, the PS and PN concentrations in the control were maintained at stable levels in the absence of CeO_2_ NPs. When the CeO_2_ NPs were added into the reactor, more PS were produced to reunite the dispersed NPs, with a gradual increase from 65.4 ± 3.6 mg/g VSS to 86.0 ± 4.2 and 93.8 ± 3.7 mg/g VSS in the R1 and R5 treatments, respectively. The PN concentration in the R5 treatment, which was much higher than that in the control (85.8 ± 3.3 mg/g VSS) or the R1 treatment (104.6 ± 3.6 mg/g VSS), increased to 136.2 ± 3.7 mg/g VSS, which indicated that different concentrations of CeO_2_ NPs had different degrees of influence on EPS production in AGS. In the second stage, we stopped adding CeO_2_ NPs and increased the influent COD concentration. As shown in Figure 3, the EPS concentrations were slightly raised in the control due to the increased influent COD. No more CeO_2_ NPs were added in the R1 and R5 treatments, and the PS and PN concentrations within the EPS significantly decreased, eventually being only slightly higher than in the control. The results showed that the different impacts of CeO_2_ NPs on the production of EPS concentrations were not irreversible.

### 3.2. Influence of CeO_2_ NPs on the LB-EPS and TB-EPS in AGS

**Figure 4:**
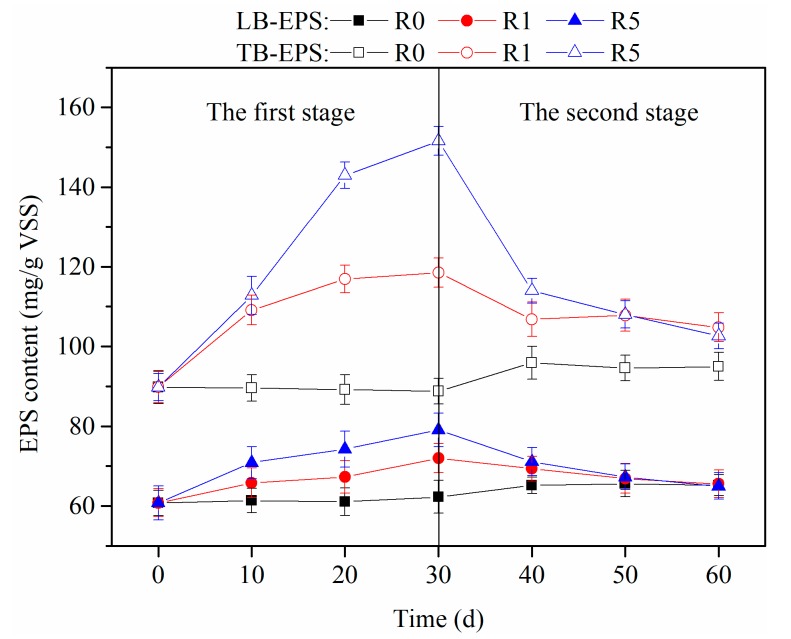
Effects of cerium oxide nanoparticles (CeO_2_ NPs) on the production of stratified extracellular polymeric substances (EPS) in aerobic granular sludge (AGS).

The EPS can be divided into LB-EPS and TB-EPS, which form two stratified protective barriers. The production of LB-EPS and TB-EPS were examined during the 60-day experiment to explore the characteristics of AGS after exposure to CeO_2_ NPs, with the results being shown in Figure 4.

The production of LB-EPS and TB-EPS displayed the same varying tendency, rising in the first stage as the CeO_2_ NP concentration increased and reducing to the levels in the control in the second stage. In the first stage, the LB-EPS concentration gradually increased from 62.3 ± 4.3 to 79.2 ± 2.8 mg/g VSS, while the TB-EPS concentration increased from 88.8 ± 3.4 to 151.6 ± 3.1 mg/g VSS. The TB-EPS concentration was significantly higher than the LB-EPS concentration as the CeO_2_ NP concentration increased. The results indicated that the CeO_2_ NPs had different degrees of influence on the distinct AGS layers. The sum of the LB-EPS and TB-EPS concentrations expressed the production of total EPS. The EPS concentration in AGS increased by 26.04% and 52.67% when exposed to 1 and 5 mg/L CeO_2_ NPs, respectively, for 30 days, as compared with the control. This result showed that the CeO_2_ NPs positively promoted the production of EPS. The enhanced EPS production served to maintain the stability of AGS, preventing CeO_2_ NP toxicity [28]. Although microorganisms could gradually adapt to the harsh environments in which NPs persisted for a long time [25], the slight increase in EPS concentrations reflected the process of microorganisms adjusting to the environment and the potential influence on the microbial community in the presence of CeO_2_ NPs, because microbial cells control the production of EPS.

In the second stage, no more CeO_2_ NPs were added in the R1 and R5 treatments, and the influent COD concentration was increased. The LB-EPS concentrations in the R1 and R5 treatments decreased to 65.6 ± 4.4 and 65.0 ± 3.1 mg/g VSS, respectively, which were similar to the concentration in the control. The TB-EPS concentrations reduced sharply, finally being only slightly higher than in the control. The results showed that the TB-EPS concentrations were more sensitive than LB-EPS concentrations to the CeO_2_ NPs, and they could recover when nanomaterials were no longer added. Nevertheless, unlike LB-EPS which were basically the same in three reactors, the TB-EPS concentrations that were produced by AGS in the R1 and R5 treatments were slightly higher than in the control after 30 days in the absence of CeO_2_ NPs, due to the different roles of the two EPS layers.

**Figure 5:**
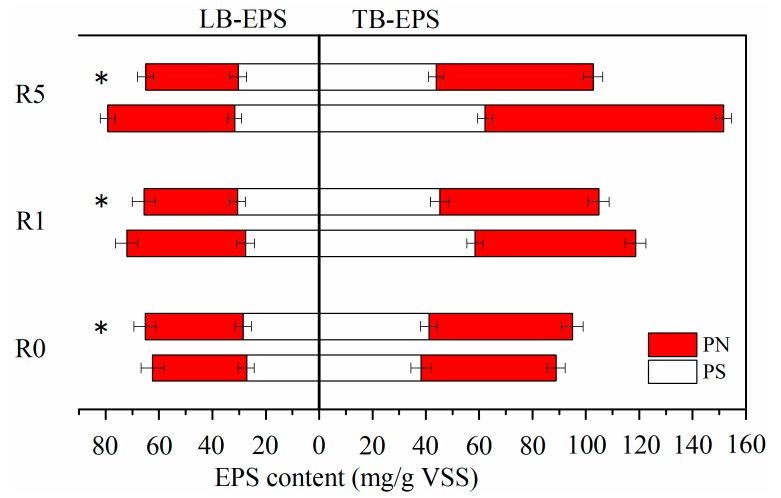
Effects of cerium oxide nanoparticles (CeO_2_ NPs) on the polysaccharide (PS) and protein (PN) concentrations in stratified extracellular polymeric substances (EPS) in aerobic granular sludge (AGS) on the 30th day. (* were on the 60th day).

To further describe the changes in EPS, the PN and PS concentrations in the LB-EPS and TB-EPS were measured and they are shown in Figure 5. On the 30th day of the R1 treatment, the PN concentration in LB-EPS and TB-EPS increased to 126.57% and 118.60% of the control value, respectively, while the PS concentrations were 1.14% and 53.14% higher than the control value, respectively. In the R5 treatment, the PN concentration in the LB-EPS and TB-EPS increased to 135.31% and 176.43% of the control value, respectively, while the PS concentrations were 16.27% and 63.02% higher than the control value, respectively. When exposed to CeO_2_ NPs, there were varying degrees of responses in the PN and PS concentrations due to their different functions in the protecting cells. The increased PS concentrations could agglomerate the NPs, creating a reticular protective layer, which protected the microbial cells against the harsh environment [29]. The increased PN concentrations in EPS may be the last barrier preventing the CeO_2_ NPs touching cell membranes or even entering cells, and therefore maintaining the microbial physicochemical and physiological properties [30].

On the 60th day, the PS and PN concentrations in LB-EPS and TB-EPS slightly increased in the control due to the increased influent COD. In the R1 and R5 treatments, because there was no addition of external CeO_2_ NPs, the PS and PN concentrations in the stratified EPS recovered to different degrees. In the R1 and R5 treatments, the PS concentrations in LB-EPS were 30.6 ± 3.0 and 30.3 ± 3.3 mg/g VSS (28.4 ± 3.1 in R0), and 45.3 ± 3.6 and 43.9 ± 3.0 mg/g VSS (41.2 ± 3.1 in R0) in TB-EPS. Meanwhile, the concentrations of PN were 35.0 ± 4.4 and 34.7 ± 3.1 mg/g VSS (36.8 ± 4.1 in R0) in LB-EPS, and 59.5 ± 4.1 and 58.8 ± 3.6 mg/g VSS (53.8 ± 4.1 in R0) in TB-EPS. The results showed that the PS concentrations that were secreted by AGS in the R1 and R5 treatments were basically the same as in the control (R0), because no excess LB-EPS was needed to prevent the effects of external NPs. However, the TB-EPS concentrations in the R1 and R5 treatments were still slightly higher than that in the control, which was due to the presence of some residual CeO_2_ NPs in the inner layers.

LB-EPS and TB-EPS were examined by LC-OCD on the 30th and 60th days of the experiment to characterize the effects of CeO_2_ NPs on the components of EPS in AGS, with the results shown in Table 2. The DOC concentration in LB-EPS and TB-EPS increased as the CeO_2_ NP concentration increased. When exposed to CeO_2_ NPs, more organic substances were produced by AGS for protection against external hazardous NPs. The DOC concentrations in LB-EPS and TB-EPS increased from 26.80 to 33.96 mg C/g VSS and 39.86 to 72.86 mg C/g VSS. High molecular weight (HMW) biopolymers played different roles in LB-EPS and TB-EPS. In LB-EPS, the biopolymers were used to aggregate the CeO_2_ NPs as they were added; however, in TB-EPS, they formed protective layers on the surface of AGS. When compared with the other DOC components, the production of LMW neutrals in TB-EPS was enhanced after the addition of CeO_2_ NPs.

On the 60th day, despite the increased influent COD, the DOC concentrations in LB-EPS and TB-EPS were less influenced in the control than the concentrations on the 30th day. In the R1 and R5 treatments, the DOC concentrations in LB-EPS decreased from 29.20 to 26.31 and 33.96 to 28.73 mg C/g VSS (26.46 in R0), respectively, while the DOC concentrations in TB-EPS decreased to 40.47 and 42.79 mg C/g VSS (39.32 in R0), respectively. When no more CeO_2_ NPs were added in the R1 and R5 treatments, the DOC concentrations in the R1 treatment were almost the same as those in the control, while the DOC concentrations in the R5 treatment were slightly higher than those in the control, due to the greater initial addition of CeO_2_ NPs. However, the LMW concentrations in the R1 and R5 treatments were still higher than in the control, which indicated that the mechanisms of resistance to CeO_2_ NPs still existed in R1 and R5, although there was no significant impact overall.

### 3.3. The Influence of CeO_2_ NPs on Nutrient Removal in AGS

**Figure 6:**
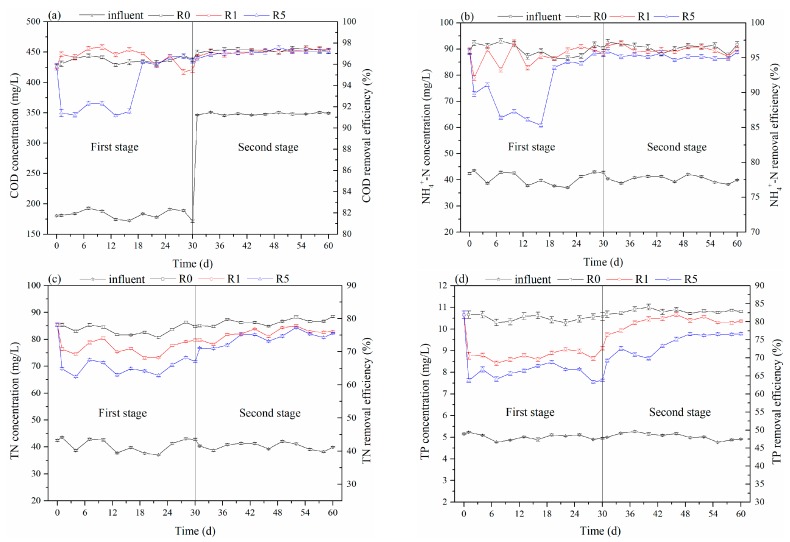
Influence of cerium oxide nanoparticles (CeO_2_ NPs) on the removal of chemical oxygen demand (COD) (**a**) and ammonia nitrogen (NH_4_^+^-N) (**b**) and total nitrogen (TN) (**c**) and total phosphorus (TP) (**d**) in the sequencing batch reactors (SBRs).

COD, NH_4_^+^-N, TN, and TP were determined every three days over the 60-day experiment to investigate the performance of the reactor. In the first stage, as shown in Figure 6, the COD and NH_4_^+^-N removal efficiencies in the R1 treatment were maintained at the same level as in the control throughout the experimental phase. However, the TN and TP removal rates in the R1 treatment were slightly influenced by the CeO_2_ NPs, and decreased to 73.47 ± 0.38% (77.65 ± 0.41% in R0) and 72.66 ± 0.47% (81.43 ± 1.13% in R0), respectively. The removal efficiencies of TN and TP in the R5 treatment (5 mg/L) significantly decreased to 66.99 ± 0.29% and 63.76 ± 0.31%, while the COD removal efficiency fell and then recovered quickly. Although the removal efficiency of NH_4_^+^-N in the R5 treatment was significantly reduced when CeO_2_ NPs were added, it recovered later in the first stage. Thus, when compared with the removal of organic matter that could quickly recover, the increase in the CeO_2_ NP concentration had more negative effects on TN and TP removal. When we stopped adding CeO_2_ NPs and increased the concentration of influent COD, the TN and TP removal efficiencies of in the R1 and R5 treatments increased slowly and then recovered. On the 60th day, compared to the control, the TN and TP removal efficiencies decreased by 4.55 ± 0.55% and 2.71 ± 0.58% in the R1 treatment, and 5.06 ± 0.46% and 6.20 ± 0.63% in the R5 treatment, respectively. The differences in COD, NH_4_^+^-N, TN, and TP removal efficiencies were related to the different characteristics of aerobic microorganisms, anaerobic microorganisms, and anoxic microorganisms, while the metabolic activity of aerobic microorganisms was less influenced than the anaerobic or anoxic microorganisms [28]. The growth and recovery of microorganisms requires the availability of organic compounds, and the increased COD in the second stage created a good growth environment for microorganisms, which had positive effects on the recovery of reactor performance [31]. The toxicity of CeO_2_ NPs on AGS was not permanent due to the strong resilience of the microorganisms.

## 4. Conclusions

This study systematically explained the responses of pollutant removal, and the production and components of stratified EPS in AGS when exposed to 1 and 5 mg/L CeO_2_ NP treatments, and then assessed the recovery as the influent COD increased. The main conclusions were:

- When exposed to different CeO_2_ NP concentrations, a greater production and broader distribution of EPS was observed. More LMW neutrals were produced in the TB-EPS as the CeO_2_ NP concentration increased, which formed denser protective layers. These responses could enhance and improve the formation of the stronger biofilm architecture to resist CeO_2_ NPs.
- Despite the LB-EPS and TB-EPS concentrations in the R1 and R5 treatments recovering and being similar to levels in the control when no CeO_2_ NPs were added, they were still slightly higher than in the R0, which indicated that the negative effects of CeO_2_ NPs could not be completely eliminated.
- The average COD removal efficiencies decreased due to the presence of CeO_2_ NPs, but rapidly recovered to the level that was observed in the control, whereas the removal of TN and TP was more sensitive, and decreased with the increase in CeO_2_ NP concentration. However, the TN and TP removal efficiencies slowly recovered in the absence of CeO_2_ NPs.

## Figures and Tables

**Table 1:** Operational modes of the two experimental stages.

Stage	Dosage	R0	R1	R5
First stage (1–30 d)	CeO_2_ NPs	0 mg/L	1 mg/L	5 mg/L
	COD	175 mg/L		
Second stage (31–60 d)	CeO_2_ NPs	0		
	COD	350 mg/L		

**Table 2:** Concentrations of dissolved organic carbon (DOC) components in the extracellular polymeric substances (EPS) in different stages.

EPS Fractions	Reactors	DOC (mg-C/g-VSS) ^a^	Biopolymers (mg-C/g-VSS) ^a^	Building Blocks (mg-C/g-VSS) ^a^	LMW Acids (mg-C/g-VSS) ^a^	LMW Neutrals (mg-C/g-VSS) ^a^
LB-EPS	R0 *	26.80	3.94	9.91	8.85	4.08
	R0 ^‡^	26.46	3.14	9.05	8.06	3.71
	R1 *	29.20	1.50	1.07	1.03	20.94
	R1 ^‡^	26.31	2.70	10.22	8.12	4.68
	R5 *	33.96	1.60	1.58	0.49	27.13
	R5 ^‡^	28.73	2.26	11.97	10.37	4.14
TB-EPS	R0 *	39.86	7.29	8.71	8.02	15.80
	R0 ^‡^	39.32	6.52	8.90	20.87	3.05
	R1 *	55.19	10.04	7.41	1.61	33.09
	R1 ^‡^	40.47	4.76	4.86	1.47	29.40
	R5 *	72.86	8.15	12.24	2.44	30.49
	R5 ^‡^	42.79	2.45	2.30	2.33	35.07

^a^ Measured by the LC-OCD system, * data on the 30th day (the first stage), ^‡^ data on the 60th day (the second stage).
